# The Clinical Relevance and Functional Implications of Thymosin Beta-10 in Glioma

**DOI:** 10.1155/2023/5517445

**Published:** 2023-11-09

**Authors:** Weimin Li, Jinliang Chen, Chengwei Xiang, Yong Long, Ke Wu, Juan Li

**Affiliations:** ^1^Department of Neurosurgery, Suining Central Hospital, Suining 629000, China; ^2^Department of Neurosurgery, Xichang People's Hospital, Xichang 615000, China; ^3^Department of Pharmacy, Suining Central Hospital, Suining 629000, China

## Abstract

Glioma is a highly aggressive form of brain cancer characterized by limited treatment options and poor patient prognosis. In this study, we aimed to elucidate the oncogenic role of thymosin beta-10 (TMSB10) in glioma through comprehensive analyses of patient data from the TCGA and GTEx databases. Our investigation encompassed several key aspects, including the analysis of patients' clinical characteristics, survival analysis, in vitro and in vivo functional experiments, and the exploration of correlations between TMSB10 expression and immune cell infiltration. Our findings revealed a significant upregulation of TMSB10 expression in glioma tissues compared to normal brain tissues, with higher expression levels observed in tumors of advanced histological grades. Moreover, we observed positive correlations between TMSB10 expression and patient age, while no significant association with gender was detected. Additionally, TMSB10 exhibited marked elevation in gliomas with wild-type IDH and noncodeletion of 1p/19q. Survival analysis indicated that high TMSB10 expression was significantly associated with worse overall survival, disease-specific survival, and progression-free survival in glioma patients. Functionally, knockdown of TMSB10 in glioma cells resulted in reduced cellular growth rates and impaired tumor growth in xenograft models. Furthermore, our study revealed intriguing correlations between TMSB10 expression and immune cell infiltration within the tumor microenvironment. Specifically, TMSB10 showed negative associations with plasmacytoid dendritic cells (pDC) and *γδ* T cells (Tgd), while displaying positive correlations with neutrophils and macrophages. These findings collectively provide valuable insights into the oncogenic properties of TMSB10 in glioma, suggesting its potential as a therapeutic target and a biomarker for patient stratification.

## 1. Introduction

Glioma, a devastating and highly aggressive brain tumor originating from glial cells, represents the majority of malignant brain tumors, with glioblastoma being the most common and aggressive subtype [1]. Gliomas pose significant challenges due to their infiltrative nature, resistance to conventional therapies, and limited treatment options, leading to poor patient prognosis [2]. Consequently, there is an urgent demand to identify novel molecular targets as well as biomarkers to improve glioma patient outcome [3].

Thymosin beta-10 (TMSB10), a small actin-binding protein, plays essential roles in cellular processes including cytoskeleton organization, cell migration, and angiogenesis [4, 5]. While primarily recognized for its physiological functions in cell motility and wound healing, emerging evidence suggests its involvement in tumorigenesis and tumor progression. Previous studies have implicated TMSB10 in various cancer types, including breast, colorectal, lung, and renal cancers [6–10]. However, the specific role of TMSB10 in glioma remains largely unexplored.

The primary objective of this study is to explore the oncogenic function of TMSB10 in glioma. We aim to elucidate the expression pattern of TMSB10 in glioma tissues compared to normal brain tissues and evaluate its clinical relevance in patient outcomes. Additionally, we seek to comprehensively understand the functional implications of TMSB10 in glioma through a combination of in vitro and in vivo experiments. Furthermore, recognizing the growing importance of the tumor microenvironment and immunotherapy in glioma treatment, we aim to explore the correlations between TMSB10 expression and immune cell infiltration.

## 2. Methods

### 2.1. Online Dataset and Immune Infiltration Analysis

The data utilized in this study were derived from publicly available online databases, including The Cancer Genome Atlas (TCGA) and Genotype-Tissue Expression (GTEx) [11]. The TCGA database provided comprehensive molecular and clinical information on glioma patients, while GTEx provided expression data of normal brain tissues for comparative analysis. TMSB10 expression levels in glioma tissues and normal brain tissues were extracted for further investigation. Raw counts value, fragments per million reads (FPKM), and transcripts per million mapped reads (TPM) normalized value were used for comparison and survival analysis.

To explore the correlations between TMSB10 expression and immune cell infiltration, we employed bioinformatics tools and algorithms to analyze the TCGA data. Specifically, we utilized the “Tumor Immune Estimation Resource” (TIMER) algorithm, which utilizes gene expression profiles to estimate the abundance of different immune cell populations within the tumor microenvironment [12]. This analysis provided valuable insights into the associations between TMSB10 expression and various immune cell subsets in glioma.

### 2.2. Patients' Characteristics

For the analysis of patients' characteristics, clinical and molecular data from the TCGA database were curated. Information on age, gender, IDH status, 1p/19q codeletion, and WHO grade was collected for glioma patients. These characteristics were then correlated with TMSB10 expression levels to investigate potential associations and prognostic implications.

### 2.3. Cell Culture and Knockdown

Glioma cell lines U251 and LN229, obtained from ATCC, were cultured in DMEM media supplemented with fetal bovine serum and antibiotics under standard conditions of a humidified incubator at 37°C with 5% CO_2_. To elucidate the functional implications of TMSB10, knockdown experiments were performed using shRNA specifically targeting TMSB10. The shRNAs were infected into the glioma cell lines according to the manufacturer's instructions as previously described [13]. Nontargeting shRNA was used as a control. The efficiency of knockdown was validated by assessing TMSB10 expression levels using quantitative real-time PCR (RT-qPCR) analysis.

### 2.4. RT-qPCR

Briefly, total RNA was extracted from the culture cells which included glioma cells with both normal and manipulated TMSB10 expression levels. Subsequently, this RNA was reverse transcribed into complementary DNA (cDNA) using specific primers and reverse transcriptase enzymes. The resulting cDNA served as a template for the quantitative polymerase chain reaction. In RT-qPCR, fluorescent DNA-binding dyes to the target gene were employed to monitor the amplification process in real-time.

### 2.5. MTT Experiments

To evaluate the impact of TMSB10 knockdown on cell viability and proliferation, MTT (3-(4,5-dimethylthiazol-2-yl)-2,5-diphenyltetrazolium bromide) assays were performed. Glioma cells with TMSB10 knockdown and control cells were seeded in 96-well plates at 10000 cells/well density. After incubation for 3 hours, MTT reagent was added to each well and further incubated. The viable cells converted the MTT reagent into purple formazan crystals, which were subsequently dissolved in dimethyl sulfoxide. The absorbance was measured at OD 450 nm absorbance using a microplate reader. By comparing the growth rates of the TMSB10-knockdown cells to the control groups, we assessed the influence of TMSB10 on glioma cell proliferation.

### 2.6. Xenograft Experiments Using Nude Mice

To better illustrate the in vivo effect of TMSB10 knockdown on tumor growth, xenograft experiments were conducted using nude mice as a model system. Glioma cells with TMSB10 knockdown and control cells were prepared as xenografts by subcutaneously injecting them into the flanks of nude mice (500,000 cells/mice). Nude mice were kept in a separately air-conditioned cabinet at temperatures of 24–26°C. Tumor growth was monitored regularly by measuring the tumor size using calipers. At the designated endpoint (four weeks), the mice were euthanized, and the tumors were excised for subsequent analysis [14].

### 2.7. Statistics

Statistical analyses were conducted using R package. Categorical variables related to patients' characteristics were analyzed using chi-square tests or Fisher's exact tests, while continuous variables were assessed using *t*-tests or Mann–Whitney *U* tests, as appropriate. Survival analyses were conducted using Kaplan–Meier curves, and log-rank tests were employed to assess the significance of differences in survival between patient groups. Multivariate Cox regression analysis was performed to identify independent prognostic factors. The results were reported as hazard ratios (HRs) with corresponding 95% confidence intervals (CIs). All experiments were repeated independently for at least three times, all statistical tests were two-sided, and a *p* value less than 0.05 was considered statistically significant.

### 2.8. Ethics

This study was performed in compliance with ethical standards and guidelines. The utilization of publicly available patient data from the TCGA database ensured patient confidentiality and adhered to data protection regulations. The xenograft experiments involving nude mice were conducted following the ethical guidelines for animal experimentation and received approval from the Suining Central Hospital Ethics Committee. Animal welfare and care were prioritized throughout the experimental procedures, and appropriate measures were taken to minimize suffering and distress.

## 3. Results

### 3.1. Correlations between TMSB10 and Patients' Characteristics

**Figure 1:**
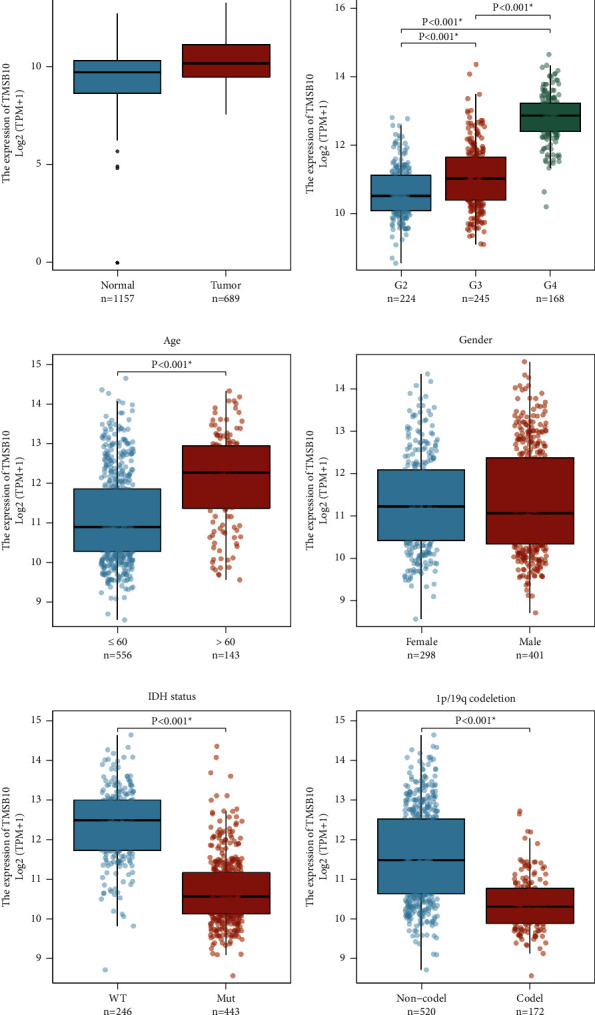
Correlations between TMSB10 expression and patients' characteristics in glioma. (a) Comparison of TMSB10 expression levels between glioma tissues and normal brain tissues using data from TCGA and GTEx databases. TMSB10 expression was significantly upregulated in glioma tissues compared to normal brain tissues. (b) Box plot illustrating the association between TMSB10 expression levels and histological grades of glioma. Higher histological grades were associated with higher TMSB10 expression levels. (c) Box plot depicting the correlation between TMSB10 expression levels and patients' age. TMSB10 expression showed a positive correlation with age in glioma patients. (d) Box plot illustrating the correlation between TMSB10 expression levels and patients' gender. No statistically significant correlation was observed between TMSB10 expression and gender. (e) Box plot demonstrating the association between TMSB10 expression levels and IDH status in glioma patients. TMSB10 showed significantly higher expression levels in glioma patients with wild-type IDH. (f) Box plot displaying the association between TMSB10 expression levels and 1p/19q codeletion in glioma patients. TMSB10 exhibited significantly higher expression levels in glioma patients with noncodeletion of 1p/19q. Data were compared using Wilcoxon rank sum test.

In this study, we found that TMSB10 was significantly higher in glioma tissues compared to normal brain tissues according to the data from TCGA and GTEx databases (Figure 1(a)). In addition, we examined the correlations between TMSB10 expression and several important clinical characteristics in glioma patients (Table 1). We observed a significant correlation between TMSB10 expression and WHO grade, a key histopathological characteristic in glioma. Patients with higher histological grades showed higher proportions of high TMSB10 expression, with grade 4 gliomas displaying the highest proportion (Figure 1(b)). This finding suggests that TMSB10 may contribute to the aggressiveness and progression of glioma, as higher-grade tumors are typically associated with poorer clinical outcomes. The correlation between TMSB10 expression and histological grade underscores the potential prognostic value of TMSB10 in predicting glioma aggressiveness and patient outcomes.

Moreover, we found a significant association between TMSB10 expression and age, with glioma patients over the age of 60 displaying a higher proportion of high TMSB10 expression compared to those aged 60 or below (Figure 1(c)). This finding suggests that TMSB10 expression may be influenced by age-related factors, indicating a potential role of TMSB10 in age-associated glioma progression. Regarding gender, our analysis did not reveal a statistically significant correlation with TMSB10 expression. The proportions of high TMSB10 expression were similar in females and males, indicating that gender-specific factors may not significantly influence TMSB10 expression in glioma (Figure 1(d)).

Furthermore, we explored the associations between TMSB10 expression and critical prognostic factors in glioma, namely, IDH status and 1p/19q codeletion. Our analysis demonstrated significant correlations between TMSB10 expression and these molecular characteristics. Glioma patients with wild-type IDH exhibited a higher proportion of high TMSB10 expression compared to those with mutant IDH (Figure 1(e)). Similarly, noncodeletion of 1p/19q was correlated with a higher proportion of high TMSB10 expression comparing to codeletion (Figure 1(f)). These findings indicate that TMSB10 may be involved in distinct molecular subtypes of glioma.

### 3.2. Survival Analysis

**Figure 2:**
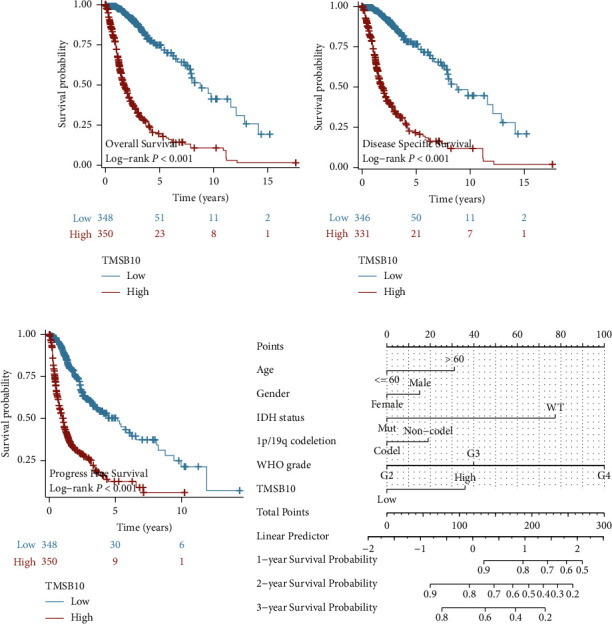
Survival analysis in glioma patients based on TMSB10 expression. (a) Kaplan–Meier curve showing the association between TMSB10 expression levels and overall survival in glioma patients. High TMSB10 expression was significantly associated with worse overall survival. (b) Kaplan–Meier curve demonstrating the association between TMSB10 expression levels and disease-specific survival in glioma patients. High TMSB10 expression was significantly associated with worse disease-specific survival. (c) Kaplan–Meier curve illustrating the association between TMSB10 expression levels and progression-free survival in glioma patients. High TMSB10 expression was significantly associated with worse progression-free survival. (d) Survival nomogram based on multivariate analysis to predict overall survival in glioma patients. The nomogram incorporates TMSB10 expression levels and other significant prognostic factors to provide individualized survival predictions.

We next evaluated the prognostic effect of TMSB10 in gliomas via Kaplan–Meier survival analyses. Accordingly, higher TMSB10 is significantly correlated with worse overall survival, cancer-specific survival, and progression-free survival (Figures 2(a)–2(c)), respectively. In addition to examining the prognostic significance of TMSB10 expression, we evaluated the impact of other clinico-pathological characteristics on patient survival.  Table 2 provides the results of the univariate and multivariate analyses, including hazard ratios (HRs) and corresponding 95% confidence intervals (CIs) for each variable.

Univariate analysis revealed age as a significant prognostic factor. Glioma patients over the age of 60 exhibited a higher HR for poor prognosis compared to those aged 60 or below (HR: 4.696, 95% CI: 3.620–6.093, *p*  <  0.001^*∗∗∗*^). This association remained statistically significant even after adjusting for other variables in the multivariate analysis, albeit with a slightly reduced HR (HR: 1.539, 95% CI: 1.130–2.097, *p*=0.006^*∗∗*^). Regarding molecular characteristics, IDH status emerged as a strong prognostic factor. Patients with mutant IDH demonstrated a significantly lower HR for poor prognosis in both univariate analysis (HR: 0.116, 95% CI: 0.089–0.151, *p*  <  0.001^*∗∗∗*^) and multivariate analysis (HR: 0.342, 95% CI: 0.224–0.523, *p*  <  0.001^*∗∗∗*^). These findings indicate that mutant IDH is associated with improved survival outcomes in glioma patients.

Furthermore, WHO grade exhibited a robust correlation with patient survival. Univariate analysis demonstrated that higher histological grades were associated with increased HRs for poor prognosis. G3 gliomas displayed an HR of 2.967 (95% CI: 1.986–4.433, *p*  <  0.001^*∗∗∗*^), while grade 4 (G4) gliomas exhibited the highest HR of 18.600 (95% CI: 12.448–27.794, *p*  <  0.001^*∗∗∗*^). In the multivariate analysis, G3 and G4 grades remained significantly associated with poor prognosis, with HRs of 1.741 (95% CI: 1.124–2.697, *p*=0.013^*∗*^) and 4.002 (95% CI: 2.338–6.851, *p*  <  0.001^*∗∗∗*^), respectively. Importantly, TMSB10 expression demonstrated significant prognostic value in both univariate and multivariate analyses. High TMSB10 expression was associated with a higher HR for poor prognosis compared to low TMSB10 expression. In the univariate analysis, the HR was 6.025 (95% CI: 4.487–8.089, *p*  <  0.001^*∗∗∗*^), indicating a strong association between high TMSB10 expression and worse clinical outcomes. After adjusting for other variables in the multivariate analysis, the HR remained significant, although with a slightly reduced magnitude (HR: 1.647, 95% CI: 1.058–2.564, *p*=0.027^*∗*^).

These results underscore the prognostic significance of age, IDH status, WHO grade, and TMSB10 expression in glioma patients. Age over 60, wild-type IDH status, higher histological grades (G3 and G4), and high TMSB10 expression were all associated with poorer survival outcomes. Therefore, we further established an overall survival nomogram to help predict overall survival of glioma patients using the abovementioned variables (Figure 2(d)), highlighting their potential utility in patient stratification and treatment decision-making.

### 3.3. *In Vitro* and *In Vivo* Experiments to Test the Glioma-Related Function of TMSB10

**Figure 3:**
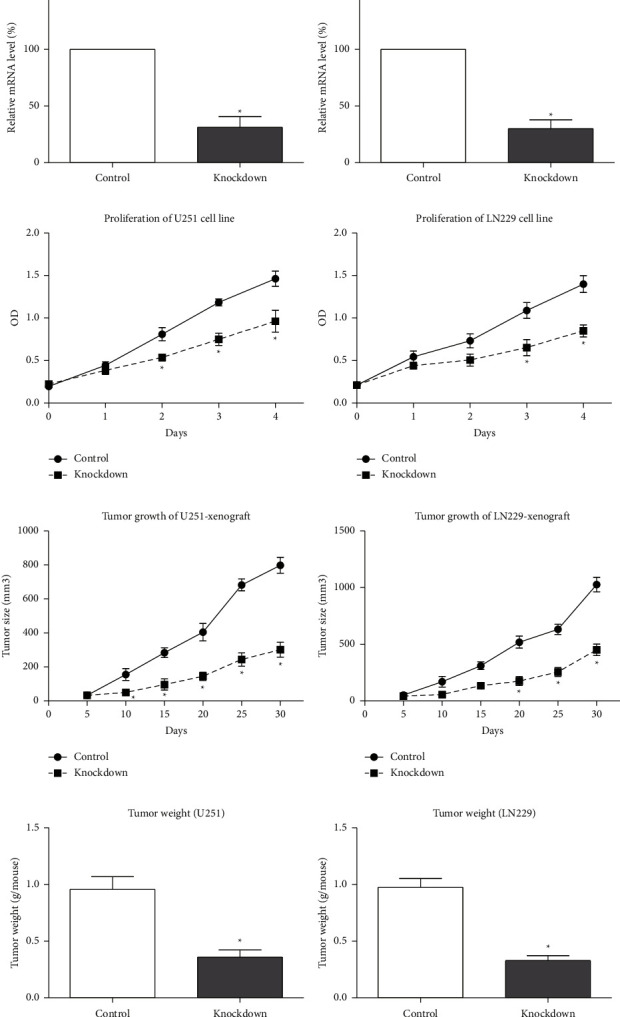
In vitro and in vivo experiments to test the glioma-related function of TMSB10. (a, b) RT-qPCR assay results showing the knockdown efficiency of TMSB10 in two glioma cells (U251 and LN229). (c, d) MTT assay results showing the growth rates of TMSB10-knockdown glioma cells (U251 and LN229) compared to control groups. TMSB10-knockdown cells exhibited significantly slower growth rates. (e, f) Representative images of xenograft tumors derived from TMSB10-knockdown glioma cells and control groups. Tumors derived from TMSB10-knockdown cells showed significantly impaired growth compared to control tumors. (g, h) Bar graph illustrating the quantification of tumor volume based on xenograft experiments. Tumors derived from TMSB10-knockdown cells exhibited significantly reduced growth compared to control tumors.

To validate the clinical findings and further investigate the functional implications of TMSB10 in glioma, we conducted in vitro and in vivo experiments. In the in vitro experiments, we utilized the U251 and LN229 glioma cell lines to evaluate the effect of TMSB10 knockdown on cellular growth rates. After validating the knockdown efficiencies (Figures 3(a) and 3(b)), MTT assays demonstrated a significant reduction in the growth rates of TMSB10-knockdown cells compared to the control groups (Figures 3(c) and 3(d)).

To assess the impact of TMSB10 knockdown on tumor growth in vivo, we performed xenograft experiments using nude mice. Glioma cells with TMSB10 knockdown and control cells were subcutaneously injected into the flanks of nude mice. The growth of xenograft tumors established by TMSB10-knockdown cells was significantly impaired compared to the control group (Figures 3(e) and 3(f)). These findings further support the oncogenic role of TMSB10 in glioma and provide evidence of its involvement in tumor growth and progression (Figures 3(g) and 3(h)).

### 3.4. Correlations between TMSB10 and Immune Cell Infiltration

**Figure 4:**
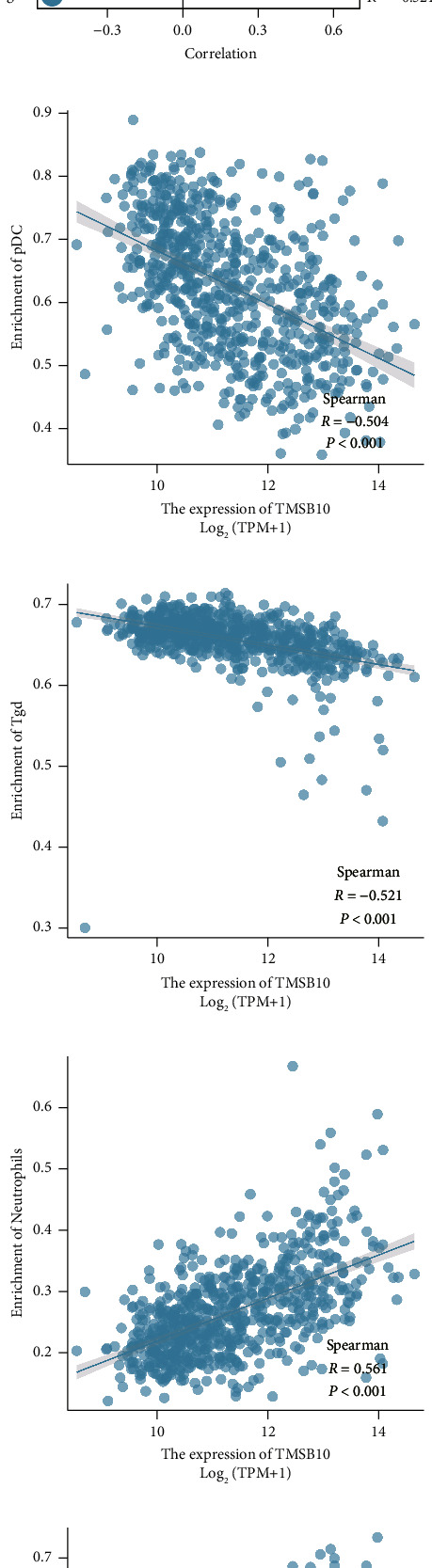
Correlations between TMSB10 expression and immune cell infiltration in glioma. (a) The correlations between TMSB10 expression and different immune cell types in the glioma microenvironment. (b) Scatter plot showing the negative correlation between TMSB10 expression and plasmacytoid dendritic cells (pDC) infiltration in glioma. (c) Scatter plot depicting the negative correlation between TMSB10 expression and *γδ* T cells (Tgd) infiltration in glioma. (d) Scatter plot illustrating the positive correlation between TMSB10 expression and neutrophil infiltration in glioma. (e) Scatter plot showing the positive correlation between TMSB10 expression and macrophage infiltration in glioma.

Given the growing interest in immunotherapy targeting glioma, we investigated the associations between TMSB10 expression and immune cell infiltration within the tumor microenvironment. Utilizing bioinformatics analysis, we explored the relationships between TMSB10 expression and immune cell populations based on TCGA data. Our analysis revealed significant correlations between TMSB10 expression and specific immune cell subsets in glioma (Figure 4(a)). For example, TMSB10 expression showed negative associations with plasmacytoid dendritic cells (pDC) and *γδ* T cells (Tgd) (Figures 4(b) and 4(c)). Conversely, TMSB10 expression exhibited positive correlations with neutrophils and macrophages (Figures 4(d) and 4(e)). These findings suggest that TMSB10 may modulate the immune microenvironment in glioma, potentially influencing immune cell recruitment and the tumor immune response.

## 4. Discussions

Glioma is a devastating type of brain tumor with poor prognosis and limited treatment options. In this study, we investigated the oncogenic role of TMSB10 in glioma and its potential as a prognostic biomarker. The upregulation of TMSB10 in glioma tissues compared to normal brain tissues provides valuable insights into its involvement in glioma pathogenesis. TMSB10 belongs to the family of *β*-thymosins, which are involved in various cellular processes, including actin dynamics, cell motility, and cytoskeletal organization [5, 15]. The physiological functions of TMSB10 in the brain are not fully understood, but its upregulation in glioma suggests its potential contribution to tumor development and progression [16]. The positive correlation between TMSB10 expression and advanced histological grades indicates its involvement in glioma aggressiveness and malignant transformation [17]. Higher-grade gliomas are characterized by increased cell proliferation, invasion, and angiogenesis, leading to worse clinical outcomes [18]. Therefore, the association between TMSB10 expression and higher histological grades suggests its potential as a marker of tumor aggressiveness. Age is an important factor in glioma development and prognosis. In our study, we observed a positive correlation between TMSB10 expression and age, with higher TMSB10 levels in glioma patients over the age of 60. Age-related changes in the tumor microenvironment and immune system may contribute to the increased TMSB10 expression observed in older glioma patients. Aging is associated with chronic inflammation and immunosenescence, which create a favorable environment for tumor growth and progression [19, 20]. TMSB10 may play a role in the age-related changes within the tumor microenvironment, potentially promoting glioma progression in older patients. Further studies are needed to elucidate the underlying mechanisms linking TMSB10 expression, age, and glioma pathogenesis.

The correlation between TMSB10 expression and molecular characteristics such as IDH status and 1p/19q codeletion highlights the potential molecular subtypes in glioma associated with TMSB10 dysregulation. IDH mutations and 1p/19q codeletion are key genetic alterations in glioma and have important implications for patient prognosis and treatment. Our findings revealed higher TMSB10 expression in glioma patients with wild-type IDH and noncodeletion of 1p/19q. Wild-type IDH gliomas often exhibit more aggressive behavior and poorer prognosis compared to mutant IDH gliomas [21]. The association between TMSB10 expression and wild-type IDH suggests that TMSB10 may be involved in the molecular pathways associated with aggressive glioma subtypes. Similarly, the association between TMSB10 expression and noncodeletion of 1p/19q further supports its potential role in distinct molecular subtypes of glioma. The underlying mechanisms linking TMSB10 dysregulation and these molecular characteristics require further investigation to better understand their functional and clinical implications.

Survival analysis revealed that high TMSB10 expression was significantly correlated to worse clinical outcomes in glioma patients. The multivariate analysis confirmed the independent prognostic value of TMSB10 expression after adjusting for other clinico-pathological characteristics. This suggests that TMSB10 expression may serve as a valuable prognostic biomarker in glioma, aiding in patient stratification and treatment decision-making. The development of a survival nomogram based on the multivariate analysis provides a practical tool for predicting overall survival in glioma patients. The inclusion of TMSB10 expression in the nomogram enhances its prognostic accuracy and may contribute to individualized treatment approaches.

Functional experiments using in vitro and in vivo models demonstrated the functional significance of TMSB10 in glioma. TMSB10 knockdown led to reduced glioma cell proliferation in vitro, indicating its role in promoting tumor growth. Furthermore, the impaired tumor growth observed in xenograft models with TMSB10-knockdown cells suggests that TMSB10 contributes to glioma progression in vivo. These findings provide experimental evidence supporting the oncogenic role of TMSB10 in glioma and suggest that targeting TMSB10 may represent a potential therapeutic strategy for glioma treatment. The tumor microenvironment plays a crucial role in tumor development and response to therapy [22]. In our study, we investigated the correlations between TMSB10 expression and immune cell infiltration within the glioma microenvironment. TMSB10 expression showed negative associations with plasmacytoid dendritic cells (pDC) and *γδ* T cells (Tgd), while positive correlations were observed with neutrophils and macrophages. These findings suggest that TMSB10 may influence the immune microenvironment in glioma, potentially modulating immune cell recruitment and function. The interplay between TMSB10 expression and immune cell infiltration highlights the complex interactions within the tumor microenvironment and may have implications for immunotherapeutic approaches targeting glioma [23].

While our study provides valuable insights into the oncogenic role of TMSB10 in glioma, there are several limitations that should be acknowledged. First, our findings are based on retrospective analysis using publicly available datasets, which may introduce inherent biases. Prospective studies with larger patient cohorts and independent validation are warranted to confirm our results. Second, the functional experiments performed in this study focused on in vitro cell proliferation and in vivo tumor growth assays. Further investigation is needed to explore the underlying molecular mechanisms through which TMSB10 promotes glioma progression, such as its impact on cell migration, invasion, and angiogenesis. Another limitation is the lack of mechanistic insights into the correlations between TMSB10 expression and patients' characteristics. The exact molecular pathways and signaling networks involving TMSB10 in glioma remain largely unknown. Future studies should aim to elucidate the molecular mechanisms underlying TMSB10-mediated oncogenic effects, including its interaction with other key molecules and pathways in glioma development and progression. Furthermore, the correlations between TMSB10 expression and immune cell infiltration, as well as its potential impact on the tumor immune microenvironment, warrant further investigation. Understanding the intricate crosstalk between TMSB10 and the immune system in glioma could provide insights into its potential as a therapeutic target for immunotherapy approaches. In terms of clinical applications, the prognostic value of TMSB10 expression should be evaluated in the context of other established prognostic markers and integrated into comprehensive prognostic models.

## 5. Conclusions

Our study provides compelling evidence for the oncogenic role of TMSB10 in glioma. TMSB10 expression was upregulated in glioma tissues and correlated with advanced histological grades, age, wild-type IDH status, and noncodeletion of 1p/19q. High TMSB10 expression was associated with worse clinical outcomes, independent of other clinical characteristics. Functional experiments supported the oncogenic function of TMSB10 in glioma cell proliferation and tumor growth. Additionally, TMSB10 expression exhibited correlations with immune cell infiltration, suggesting its potential influence on the tumor immune microenvironment. Collectively, our findings highlight TMSB10 as a promising therapeutic target and prognostic biomarker in glioma, opening avenues for further research into the underlying mechanisms and potential clinical applications of targeting TMSB10 in this devastating disease.

## Figures and Tables

**Table 1:** Correlations between TMSB10 level and characteristics of glioma patients.

Characteristics	Low expression of TMSB10	High expression of TMSB10	*P* value
*n*	349	350	
Age, *n* (%)			<0.001^*∗∗∗*^
≤60 yrs	319 (45.6%)	237 (33.9%)	
>60 yrs	30 (4.3%)	113 (16.2%)	
Gender, *n* (%)			0.234
Female	141 (20.2%)	157 (22.5%)	
Male	208 (29.8%)	193 (27.6%)	
IDH status, *n* (%)			<0.001^*∗∗∗*^
WT	20 (2.9%)	226 (32.8%)	
Mut	326 (47.3%)	117 (17%)	
1p/19q codeletion, *n* (%)			<0.001^*∗∗∗*^
Noncodel	199 (28.8%)	321 (46.4%)	
Codel	150 (21.7%)	22 (3.2%)	
WHO grade, *n* (%)			<0.001^*∗∗∗*^
G2	168 (26.4%)	56 (8.8%)	
G3	134 (21%)	111 (17.4%)	
G4	2 (0.3%)	166 (26.1%)	

**Table 2:** Univariate and multivariate overall survival analyses.

Characteristics	Total (*N*)	Univariate analysis		Multivariate analysis	
		Hazard ratio (95% CI)	*P* value	Hazard ratio (95% CI)	*P* value
Age	698				
≤60 yrs	555	Reference		Reference	
>60 yrs	143	4.696 (3.620–6.093)	<0.001^*∗∗∗*^	1.539 (1.130–2.097)	0.006^*∗∗*^
Gender	698				
Female	297	Reference		Reference	
Male	401	1.250 (0.979–1.595)	0.073	1.234 (0.937–1.625)	0.134
IDH status	688				
WT	246	Reference		Reference	
Mut	442	0.116 (0.089–0.151)	<0.001^*∗∗∗*^	0.342 (0.224–0.523)	<0.001^*∗∗∗*^
1p/19q codeletion	691				
Noncodel	520	Reference		Reference	
Codel	171	0.225 (0.147–0.346)	<0.001^*∗∗∗*^	0.768 (0.459–1.286)	0.316
WHO grade	636		<0.001^*∗∗∗*^		
G2	223	Reference		Reference	
G3	245	2.967 (1.986–4.433)	<0.001^*∗∗∗*^	1.741 (1.124–2.697)	0.013^*∗*^
G4	168	18.600 (12.448–27.794)	<0.001^*∗∗∗*^	4.002 (2.338–6.851)	<0.001^*∗∗∗*^
TMSB10	698				
Low	348	Reference		Reference	
High	350	6.025 (4.487–8.089)	<0.001^*∗∗∗*^	1.647 (1.058–2.564)	0.027^*∗*^

## Data Availability

All data generated or analyzed during this study are included in this published article.
